# Complex Coronary Artery Bypass Grafting: Intraoperative Challenges and Surgical Strategies in Contemporary Practice

**DOI:** 10.3390/jcm15072775

**Published:** 2026-04-07

**Authors:** Ahmed Osman, Karim Elrakhawy, Dominique Shum-Tim

**Affiliations:** 1 Faculty of Medicine, University of Montreal, 850 Boul. de Maisonneuve E, Montreal, QC H2L 4L8, Canada; 2 McGill University Health Centre, Division of Cardiac Surgery, Department of Surgery, McGill University Faculty of Medicine, Montreal, QC H3A 1A3, Canada

**Keywords:** coronary artery bypass grafting, intramural coronary, embedded coronary, myocardial bridge, diffuse coronary disease, calcified coronary, coronary endarterectomy, small target vessel, right ventricular injury, ventricular perforation

## Abstract

**Background**: Contemporary coronary artery bypass grafting (CABG) is often performed in patients with diffuse atherosclerosis, severe calcification, prior percutaneous coronary intervention (PCI), and fragile myocardium, creating intraoperative scenarios that can compromise target selection, anastomotic quality, and completeness of revascularization. We synthesize operative strategies and outcomes across five predefined “complex CABG” scenarios. **Methods**: A focused literature review was performed targeting intraoperative CABG challenges in adult patients. Two reviewers independently screened titles/abstracts and selected studies describing operative details, technical considerations, or outcomes relevant to (1) intramyocardial/embedded coronaries, (2) severely calcified or diffuse disease requiring reconstruction, (3) small-caliber targets/flow-limited grafting, (4) iatrogenic right ventricular (RV) injury, and (5) failed PCI/stent-related surgical management. Disagreements were resolved through discussion and consensus. **Results**: Thirty core publications were synthesized across five complex intraoperative CABG scenarios (intramural/embedded coronaries *n* = 7; calcified/diffuse disease *n* = 7; small-caliber/flow-limited targets *n* = 7; iatrogenic RV injury *n* = 5; failed PCI/stent-related management *n* = 5). Intramural/embedded targets: reported intramyocardial LAD prevalence ranged from 2.2–13%, and studies emphasized structured localization strategies with a small but real risk of ventricular injury depending on technique. Severely calcified/diffuse disease: reconstructive approaches (endarterectomy, patch angioplasty, long-segment LAD reconstruction) were used to create graftable beds when standard anastomosis was not feasible, with series reporting acceptable early mortality and generally high early-to-midterm patency when paired with planned antithrombotic and imaging follow-up strategies. Small-caliber targets: vessel size alone did not preclude durable grafting when flow was optimized, with evidence supporting flow-augmenting designs (e.g., sequential grafting) and intraoperative flow verification to reduce low-flow failure in limited runoff beds. Iatrogenic RV injury: bailout techniques prioritized rapid hemostasis while preserving LAD/graft patency using buttressed closure concepts designed for constrained exposure and ongoing bleeding risk. Failed PCI/stent-related pathology: long stented segments shifted operative planning from distal target selection to target reconstruction (stentectomy/endarterectomy with long-segment LAD reconstruction), with angiographic follow-up cohorts demonstrating feasible revascularization but variable patency by territory and lesion extent. **Conclusions**: Complex CABG is best approached as structured, anatomy-driven problem-solving: deliberate target localization, creation of a graftable bed when needed, flow-augmenting graft design, and predefined bailout options. Standardized comparative studies are needed to define optimal strategies across these common clinically important scenarios.

## 1. Introduction

Coronary artery bypass grafting (CABG) remains a cornerstone of surgical revascularization, being the most performed cardiac operation in the world [1]. CABG continues to provide symptom relief and prognostic benefit in appropriately selected patients with advanced coronary artery disease. However, the operative reality of CABG is evolving. Contemporary patients often present with diffuse coronary disease, severe calcification, prior percutaneous coronary interventions (PCIs), and more fragile myocardial tissue, which increase operative complexity and narrow the margin for error [2,3,4,5]. As a result, surgeons are more frequently required to deviate from “standard” bypass strategies to achieve safe, durable, and complete revascularization. These complex operative scenarios are no longer the exception but an increasingly common feature of routine practice.

Moreover, these complex operative CABG scenarios have meaningful clinical consequences. Technical difficulty during CABG is not merely a question of operative time, but directly influences surgical outcomes, including target vessel exposure, the feasibility of a durable arteriotomy, anastomotic quality, myocardial protection, bleeding risk, completeness of revascularization, and the likelihood of early graft failure [6]. Intraoperative decisions in these settings often must be made rapidly and are primarily driven by coronary anatomy and myocardial physiology rather than by standardized, procedure-specific algorithms. Contemporary literature does not explicitly address many of these intraoperative challenges. Instead, relevant evidence is dispersed across small technique series and heterogeneous observational reports. As a result, operative approaches vary widely between surgeons and institutions, and the same anatomic problem may be managed using different strategies [7].

In this review, we define “complex CABG” as intraoperative scenarios that increase the risk of technical failure, incomplete revascularization, or major complications that commonly require modified techniques. Five situations are emphasized because they are both increasingly encountered and clinically impactful: (1) intramural or deeply embedded coronary arteries, (2) severely calcified or diffuse coronary disease, (3) small-caliber target vessels, (4) iatrogenic right ventricular perforation and (5) the surgical management of failed PCI.

The aim of this paper is to review current evidence and expert operative practice across these five domains. The goal is to define a common approach that can help guide surgeons in the management of complex CABG scenarios.

## 2. Materials and Methods

A narrative literature review was conducted to synthesize current evidence on complex intraoperative challenges encountered during coronary artery bypass grafting (CABG). Searches were performed in PubMed, Embase, and Web of Science for articles published between 2010 and 2026 using Boolean combinations tailored to five predefined complex CABG scenarios. The search strategy combined terms related to CABG with scenario-specific keywords, including but not limited to: (“coronary artery bypass grafting” OR “CABG”) AND (“intramural coronary” OR “embedded coronary” OR “myocardial bridge”), (“diffuse coronary disease” OR “calcified coronary” OR “coronary endarterectomy”), (“small target vessel” OR “poor distal runoff”), (“right ventricular injury” OR “ventricular perforation”), and (“failed PCI” OR “stent complication” OR “bailout CABG”).

The most relevant and recent studies were prioritized to reflect contemporary surgical practice, while older landmark studies were included when necessary to capture foundational operative techniques. Only English-language studies involving adult patients were considered. Eligible studies reported intraoperative technical challenges during CABG and addressed at least one of the predefined complex scenarios: (1) intramural or deeply embedded coronary arteries, (2) severely calcified or diffuse coronary disease requiring advanced reconstructive techniques, (3) small-caliber or poor-quality target vessels, (4) iatrogenic right ventricular injury during CABG, or (5) failed percutaneous coronary intervention requiring surgical bailout. Studies focusing exclusively on non-surgical coronary interventions, pediatric populations, animal models, or non-CABG cardiac procedures were excluded. Reference lists of included articles were manually screened to ensure completeness.

A total of 2 reviewers independently screened titles and abstracts for relevance. Studies were included if they described adult patients undergoing CABG and provided operative details, technical considerations, or outcomes related to one or more of the predefined complex intraoperative scenarios. Studies were excluded if they lacked intraoperative relevance, focused solely on postoperative outcomes without surgical context, or did not address technical decision-making during CABG. Discrepancies in study selection were resolved through discussion and consensus.

A total of 39 articles were ultimately included. From each study, data were extracted regarding study design, sample size, coronary anatomy involved, intraoperative challenge encountered, surgical techniques employed and reported outcomes. Emphasis was placed on extracting operative strategies, technical adaptations, and decision-making principles applicable to complex CABG scenarios. Given the narrative nature of this review, no formal risk-of-bias assessment was performed. Instead, priority was given to studies with detailed operative descriptions and clear relevance to real-world surgical practice.

## 3. Results

**Figure 1:**
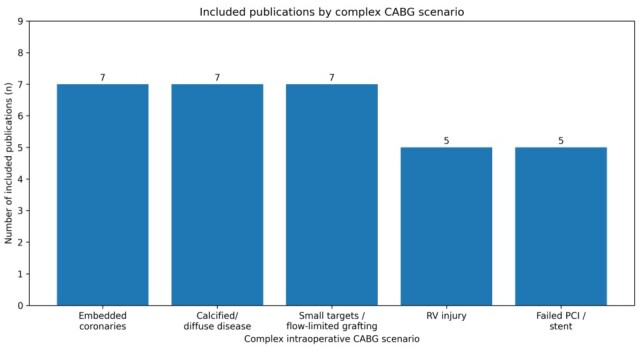
Number of included publications by complex CABG scenario.

As shown in Figure 1, of the 41 articles included in the overall review, a total of 31 core publications were selected for synthesis across five complex intraoperative CABG scenarios: intramural or deeply embedded coronary arteries (*n* = 7), severely calcified or diffuse coronary disease (*n* = 7), small-caliber target vessels and flow-limited grafting (*n* = 7), iatrogenic right ventricular (RV) injury during LAD localization or beating-heart CABG (*n* = 5), and failed percutaneous coronary intervention (PCI)/stent-related surgical management (*n* = 5). Table 1 summarizes the included studies and key findings across scenarios.

Seven studies examined intramural or deeply embedded coronary targets, including retrospective cohorts describing prevalence, localization approaches, and exposure-related complications. Reported intramyocardial LAD prevalence ranged from 2.2% to 13%. In a large series of 4102 CABG patients, probe-guided localization was used in most cases (73/92, 79%), and right ventricular injury occurred in four patients (predominantly with direct dissection), with no hospital mortality reported in the embedded-LAD subgroup. Across cohorts, most intramyocardial LAD segments were superficial (≈96%) while a minority were deeply embedded (≈4%). Technical adjuncts (epicardial Doppler flow detection and epicardial ultrasound) and minimally invasive/off-pump unroofing techniques were also described to facilitate safe target identification and anastomosis.

Six studies addressed severely calcified or diffuse coronary disease, focusing on reconstructive strategies such as coronary endarterectomy, vein-patch angioplasty, and long-segment LAD reconstruction with internal mammary grafting. In the largest endarterectomy cohort (n = 188), 30-day mortality was 1.1% with perioperative myocardial infarction in 9.0%; reported LAD patency was 91.6% early and 96.6% at 1 year, with 5-year freedom from mortality of 89.3%. A smaller vein-patch angioplasty cohort (n = 21) reported 4.8% early mortality, 5-year survival of 81%, and graft patency of 93.3% among patients who underwent follow-up imaging. In a randomized study of 40 patients with diffuse LAD disease, myocardial infarction occurred only in the long arteriotomy/long anastomosis group, while the sequential anastomosis group demonstrated greater improvement in ejection fraction and functional class. Additional series and case-based reports described saphenous vein patch reconstruction and open endarterectomy technique refinements for non-standard or heavily diseased targets.

Seven studies evaluated strategies for small-caliber target vessels and flow-limited grafting, spanning large total-arterial/off-pump cohorts, comparative analyses of adjunctive techniques for challenging targets, and transit-time flow measurement (TTFM)-based assessment of graft quality. In a total arterial off-pump series (n = 633; 2617 distal anastomoses), patency to targets 1.25 mm or smaller was 97.4% (626/643). Comparative cohort analyses reported that small (<1.5 mm) and/or heavily calcified targets required more adjunctive techniques but achieved comparable early ischemic outcomes at 1 year. Meta-analytic data suggested lower graft failure with sequential saphenous vein grafting compared with individual vein grafts (risk ratio 0.68). However, target-vessel specific failure rates were not consistently reported across studies, limiting conclusions regarding whether this difference was driven by a particular coronary territory. TTFM studies associated lower mean graft flow and higher pulsatility index with inferior patency and identified a pulsatility index greater than 5 as an independent predictor of major adverse cardiac and cerebrovascular events.

Four publications described iatrogenic RV injury during LAD localization or beating-heart CABG, primarily as technical notes and case-based reports. Repair strategies emphasized rapid hemostasis without compromising LAD or graft flow, including sandwich patch techniques and reinforced closures. In two reported beating-heart cases, the sandwich technique enabled hemostasis and hospital discharge around postoperative day 5. A detailed case of RV perforation during intramyocardial LAD revascularization described a 4 mm defect successfully repaired with preserved graft flow (22 mL/min; pulsatility index 2.2), discharge on postoperative day 7, and Canadian Cardiovascular Society class I status at 18-month follow-up.

Finally, six studies focused on operative management after failed PCI or stent-related pathology. Technical reports and series highlighted stentectomy with endarterectomy, long-segment LAD reconstruction in full-metal-jacket scenarios and planned imaging follow-up (computed tomography angiography or coronary angiography). In the largest cohort with angiographic follow-up (n = 160), overall graft patency to endarterectomized targets was 69.9%, with higher patency for LAD endarterectomy (80.0%) than non-LAD sites (64.2%); postoperative myocardial infarction occurred in 4.4%, and right coronary endarterectomy independently predicted graft failure (odds ratio 2.35). Additional case-based reports addressed rare presentations (late stent infection) and hybrid bailout strategies (intraoperative drug-coated balloon angioplasty via LAD arteriotomy).

## 4. Discussion

### 4.1. Intramyocardial LAD: Exposure and Localization

When the LAD courses intramyocardially, superficial epicardial landmarks may be absent, making it difficult to identify an appropriate bypass target. Reported prevalence varies across different studies, from 2.2% to 13%, likely reflecting differences in definitions and detection rather than a true epidemiologic discrepancy. Nonetheless, this reinforces that this scenario is encountered often enough to merit a deliberate strategy rather than improvisation [8,9,10].

When exposure relies on aggressive or blind dissection, the risk of coronary dissection, ventricular injury, and significant bleeding increases substantially during target localization [11]. As a result, contemporary techniques increasingly favor conservative, stepwise localization and controlled exposure before committing to deeper dissection. More recent studies have therefore shifted from describing the problem to outlining practical techniques to overcome it in the operating room.

A conservative, stepwise strategy begins by identifying any visible distal LAD segment and tracing it proximally to estimate the buried course. Surface landmarks, particularly the anterior interventricular groove and anterior interventricular vein, can help narrow the dissection plane and guide expected LAD emergence [9]. Palpation for calcification or a prior stent may further aid localization when present. If these maneuvers remain insufficient, retrograde probe-guided localization from the apex may be used selectively to identify the vessel, although this should be reserved for difficult cases given the risk of ventricular injury and bleeding with more invasive exposure maneuvers [9,11]. This technique-focused framework is informed by cohort data suggesting that the importance of embedded LAD anatomy lies less in mortality and more in operative complexity and exposure-related risk. In a 4102-patient on-pump CABG series, embedded LAD anatomy was associated with longer cross-clamp and bypass times without higher in-hospital mortality or worse short- to mid-term survival. However, right ventricular injury occurred predominantly with direct dissection rather than retrograde probe-guided exposure, indicating that the method of localization may influence complication risk. Accordingly, this framework refers to a structured, stepwise localization strategy rather than blind or aggressive dissection [10]. When surface-based cues and careful probing remain insufficient, the literature supports adding problem solving adjuncts rather than widening dissection. Doppler-based approaches can help detect coronary flow and guide localization when the LAD is not directly visible [12]. In the most difficult cases, intraoperative epicardial ultrasound has been described as a novel option that delineates the depth and trajectory of an intramyocardial LAD, enabling controlled exposure and anastomosis when conventional surface landmarks fail [13]. When present, a diagonal branch can also be opened and probed at its junction with the LAD to help localize the LAD course without broadening the dissection.

Even with a structured, stepwise approach, complication reports highlight the stakes of exposure. Right ventricular perforation has been described during attempts to access an intramyocardial LAD, and repair must achieve hemostasis while preserving coronary and graft patency, reinforcing why prevention through controlled localization is critical [29]. Conversely, minimally invasive off-pump unroofing illustrates that LAD caliber and graftability can be underestimated before adequate exposure, supporting deliberate unroofing rather than premature abandonment of the target [14]. Collectively, these data support a stepwise exposure strategy that prioritizes surface-based localization and reserves adjunct imaging or probe guidance for unresolved cases, minimizing preventable injury during target identification and exposure.

### 4.2. Diffuse or Calcified Disease: Reconstruction Strategies

When coronary disease is severely calcified or diffusely atherosclerotic, the primary limitation is often the lack of a suitable anastomotic target rather than conduit availability, making a standard focal arteriotomy and anastomosis unreliable or incomplete. In this context, pushing through with a short, forced anastomosis on hostile plaque risks suboptimal runoff, technical failure, and perioperative ischemic events [15]. This is why contemporary work increasingly frames these cases as a reconstruction problem where the surgeon must first create a graftable conduit segment before bypassing it [15,16,17,18,19,20,39].

At the more aggressive end of the reconstructive spectrum, long-segment coronary endarterectomy with arterial reconstruction can be used when no suitable target exists. In a large 10-year experience of diffusely diseased LAD, long arteriotomy endarterectomy followed by internal thoracic artery reconstruction achieved acceptable early safety with durable patency, supporting this strategy when conventional grafting is not feasible [15]. More extensive endarterectomy concepts have also been described, including full metal jacket type approaches, reinforcing that even aggressive long-segment plaque extraction can achieve reasonable midterm outcomes in appropriately selected patients who would otherwise face incomplete revascularization [20]. Case-based technical work further supports the feasibility of open coronary endarterectomy while emphasizing that meticulous technique and careful patient selection are central to minimizing embolization, bleeding, and early graft failure [19].

**Figure 2:**
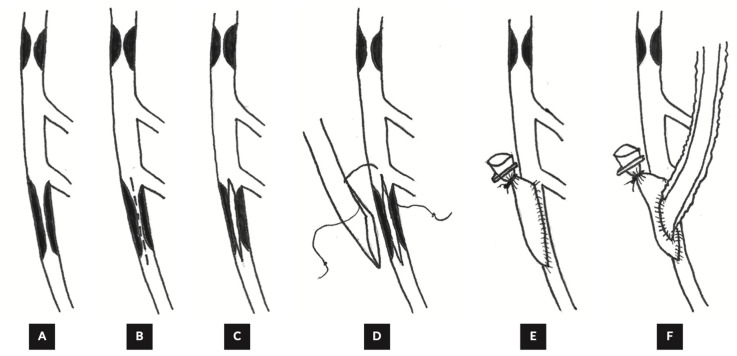
Steps in vein patch angioplasty with CABG. (**A**) LAD with segmental stenoses. (**B**) Proposed line of arteriotomy on the LAD (dashed line). (**C**) Arteriotomy splitting the plaque of the distal stenosis. (**D**) Saphenous vein anastomosis to the LAD. (**E**) Completed vein patch on the LAD. (**F**) LIMA has been anastomosed to the vein patch on the LAD [18].

At the less aggressive end, patch-based strategies can extend the anastomotic bed without extensive plaque extraction. As shown in Figure 2, Saphenous vein patch angioplasty followed by left internal thoracic artery grafting to the reconstructed left anterior descending artery has been reported with acceptable midterm outcomes and high graft patency on follow-up imaging, supporting patch reconstruction as a practical alternative when long endarterectomy is undesirable or high risk [16,18]. More recent practical series further support an internal mammary artery on patch paradigm as a stepwise solution when there is no single good LAD target, while reserving endarterectomy for cases in which severe calcification leaves no exploitable segment [18].

In summary, patch reconstruction is preferred when a graftable bed can be created without plaque extraction, whereas long-segment endarterectomy is reserved for severely calcified or diffusely occlusive LAD disease in which no exploitable target exists for conventional grafting [15,16,18].

Within reconstructive strategies, the extent of arteriotomy and anastomotic configuration also matters. In a randomized comparison of two left internal thoracic artery (LIMA) to left anterior descending (LAD) approaches for severe diffuse disease, myocardial infarction occurred only in the long arteriotomy, single extended anastomosis group, whereas no myocardial infarction was observed with sequential jump anastomoses. Moreover, the sequential approach was associated with greater improvement in left ventricular ejection fraction and functional class, suggesting a potential advantage to limiting arteriotomy length when anatomy permits [17]. These findings reinforce that diffuse, calcified LAD disease requires tailored arteriotomy planning rather than a one-size-fits-all bypass [17].

### 4.3. Small-Calibre Coronaries: Optimize Flow and Design

When coronary targets are small calibre, the primary challenge is less the technical feasibility of the anastomosis and more the postoperative hemodynamic performance of the graft. This is because small vessels often imply higher distal resistance, limited runoff, and a higher risk that the graft becomes low-flow. Additionally, small-caliber targets increase the risk of technical difficulty during anastomosis, as limited luminal size magnifies the impact of even minor imprecision or narrowing. Accordingly, small calibre should not be treated as “no bypass,” but as an indication to optimize inflow and construct design so anterograde flow and patency are preserved rather than excluding revascularization based on diameter alone.

This concept is supported by an off-pump series of sequential and composite arterial grafting to more than five coronary branches, in which angiographic patency remained high even for very small targets, including vessels at or below 1.25 mm. The size-stratified results in Table 2 further suggest that small-calibre bypass can remain durable when inflow and graft configuration are deliberately optimized [21].

The same principles apply when small-calibre targets coexist with distal coronary calcinosis. This phenotype often necessitates more complex grafting strategies to achieve complete revascularization and to maintain adequate flow across a high-resistance distal bed. In a propensity-matched cohort, vessels < 1.5 mm were more common in patients with distal calcification, who also more frequently required flow-augmenting strategies such as Y grafts and sequential grafting. Outcomes at one year remained comparable to controls, indicating that planned technical escalation can preserve complete revascularization [22]. Similar cohort experience in calcified targets shows that small calibre anastomoses are frequent and that success typically requires deliberate adaptation rather than deferring distal grafting [23].

Because graft flow is the main vulnerability in small-calibre targets, sequential grafting becomes particularly attractive when runoff is limited, as one conduit can serve multiple distal beds and thereby sustain higher overall graft flow rather than a marginal, low-flow state. Pooled evidence suggests lower graft failure with sequential vein grafts compared with individual vein grafts. However, this finding should be interpreted cautiously because comparative studies did not consistently stratify failure by coronary territory or target-vessel site. Therefore, it cannot be concluded from the available data that the higher failure rate of individual vein grafts is specific to one coronary artery alone. Rather, patency likely depends on the characteristics of the distal bed, including vessel calibre, runoff, and target quality, in addition to graft configuration. In this context, sequential grafting may be particularly beneficial when several small or flow-limited targets are revascularized with a shared conduit, whereas conduit length, number of distal anastomoses, and intraoperative flow characteristics must still be assessed carefully to avoid low-flow failure [24,25,40].

Finally, intraoperative verification is particularly important in small-calibre targets, where a technically acceptable anastomosis can still be physiologically low flow. Transit-time flow measurement has shown that low mean graft flow and an elevated pulsatility index are associated with poorer patency and higher rates of adverse events, supporting intraoperative reassessment and revision when flow parameters are borderline, especially in sequential constructs [26,27]

Taken together, small calibre targets are best managed as a flow-planning problem. Surgeons should prioritize inflow-rich arterial conduits, use sequential or composite configurations when runoff is limited, adapt the strategy in the setting of distal calcinosis, and confirm graft performance intraoperatively to reduce preventable failure.

### 4.4. Iatrogenic RV Perforation: Hemostasis Without Compromising LAD Flow

Even with careful planning, right ventricular (RV) perforation remains a high-stakes intraoperative complication during attempts to expose a deeply embedded or intracavitary LAD. Across bailout reports, a consistent operative priority emerges: achieve hemostasis while preserving LAD patency and graft flow, because an overly tight repair can control hemorrhage at the cost of distal ischemia.

**Figure 3:**
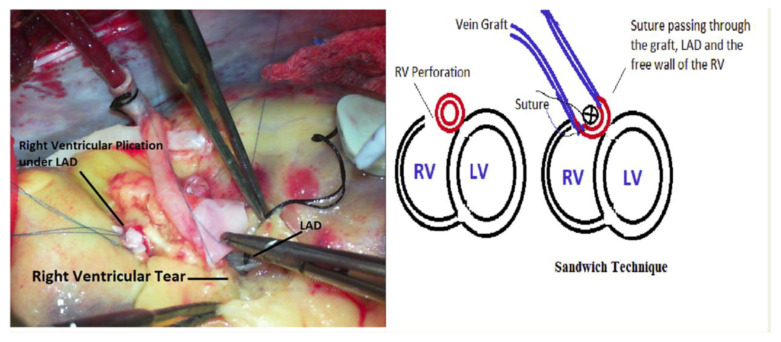
Intraoperative repair of right ventricular perforation beneath the LAD using a sandwich construct, shown in the operative photograph (**left**) and corresponding schematic illustration (**right**) [28].

One strategy addresses this with a “sandwich” construct that uses the native LAD and the bypass graft as a buttress, sealing the perforation without narrowing the target vessel or jeopardizing graft patency. This specific technique is illustrated in Figure 3 [28]. Complementary case experience emphasizes intentional suture geometry, capturing myocardium securely while keeping stitches offset from the LAD course; some authors use a small probe to preserve luminal space during tie-down, explicitly treating hemostasis and patency as linked endpoints [29]. Another technique reinforces closure using the adjacent coronary wall to distribute tension across the repair, aiming to reduce rebleeding while avoiding luminal compromise when exposure is limited and bleeding is significant [30]. Practical reports further note that this injury most often occurs during aggressive vessel localization and describe simple closure aids to control bleeding while protecting the LAD during ventriculotomy repair [31]. Finally, minimally invasive workflows underscore the same principle: limited exposure increases the consequences of both bleeding and imprecise closure, making prevention and bailout readiness integral to the operative plan [32]. In practical terms, repair should incorporate a generous segment of healthy myocardium reinforced with felt or pledgets to prevent suture line tearing. Closure must be performed without ventricular tension, ideally under cardiopulmonary bypass, to optimize hemostasis while preserving LAD patency and graft integrity.

### 4.5. Failed PCI: When Bypass Becomes Target Reconstruction

**Figure 4:**
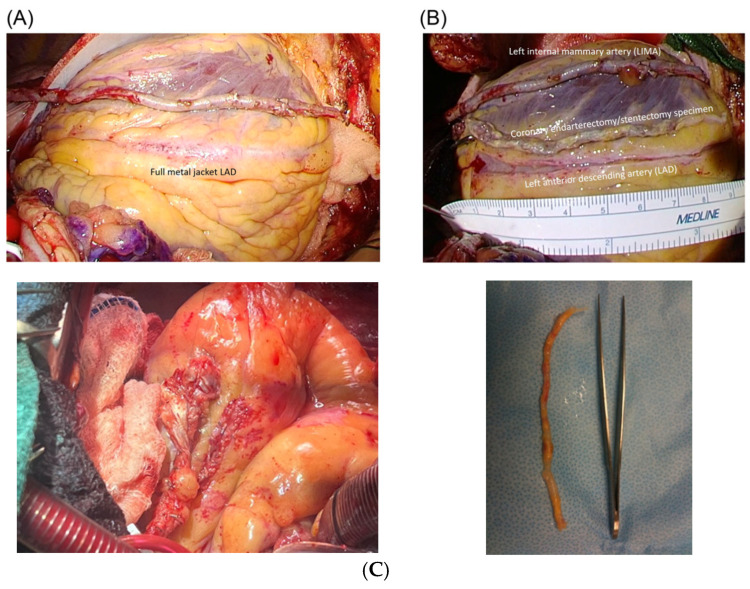
Intraoperative sequence for full-metal-jacket LAD reconstruction. (**A**) Full-metal-jacket LAD. (**B**) After stentectomy/endarterectomy. (**C**) Onlay patch reconstruction using the skeletonized IMA [33].

Prior percutaneous coronary intervention can convert a routine bypass into a target creation problem. Long stented segments, diffuse in stent restenosis, and the absence of a clean distal landing site can make a standard distal anastomosis unreliable, not because the conduit is inadequate, but because the native vessel is no longer graftable without first rebuilding a usable segment [33] as seen in Figure 4.

In this setting, the operative strategy shifts from finding a distal target to rebuilding a target, most commonly through stentectomy with endarterectomy followed by an onlay-style LAD reconstruction before grafting. The stented LAD is exposed (Figure 2A), followed by stentectomy/endarterectomy (Figure 2B) and LIMA onlay reconstruction (Figure 2C) [33]. Off-pump experience with stent removal for LAD in stent restenosis supports technical feasibility, with angiographic follow-up demonstrating a reconstructed segment that can accept mammary grafting when no suitable distal vessel is available [34,36]. Larger contemporary series of stent endarterectomy for extensive full metal jacket LAD disease similarly frame the operation as controlled reconstruction, reporting low early mortality and acceptable midterm outcomes when paired with a planned antithrombotic regimen and imaging follow-up strategy [35]. Technical how-to descriptions add further clarity by outlining a reproducible sequence for safe stentectomy, endarterectomy, and mammary onlay reconstruction in cases with no distal target, emphasizing that success depends on converting a metal-lined, noncompliant artery into a stable graftable segment rather than forcing a small distal anastomosis [33].

Not all failed PCI anatomy reflects restenosis. Rare but catastrophic late stent infection with myocardial abscess may mandate debridement, stent removal, and distal bypass, underscoring situations in which the goal shifts from revascularization alone to source control plus revascularization [37]. At the other end of the spectrum, hybrid approaches can fill gaps when distal disease would otherwise require complex reconstruction, using intraoperative angioplasty through LAD arteriotomy access to treat distal diffuse disease while still completing surgical grafting, highlighting that bailout planning can include hybrid options when anatomy is unfavorable [38].

Taken together, failed PCI should be approached as a structured decision pathway: define the stented segment and available target, select reconstruction when no distal landing site exists, and keep contingency strategies available for infection, bleeding, or distal diffuse disease.

## 5. Conclusions

Complex CABG often requires techniques beyond standard revascularization. Intramyocardial LAD, diffuse or heavily calcified disease, and small-caliber targets can limit routine distal anastomosis. These patterns often demand modified exposure and alternative reconstruction strategies. Prior PCI can also complicate target selection and graft planning. Intraoperative complications, including right ventricular injury, remain uncommon but high-impact. They require early recognition and rapid, controlled management.

Across these scenarios, success depends on strong preoperative assessment. Coronary anatomy, vessel quality, and myocardial territory should guide a clear operative plan. The plan should include predefined bailout options. Intraoperatively, meticulous dissection and atraumatic handling are critical. The goal is consistent: safe revascularization with durable graft function. As the CABG population becomes more complex, structured decision-making becomes increasingly important. Future work should compare strategies using standardized definitions and clinically meaningful outcomes.

## 6. Limitations

A main limitation of this review is its narrative design, which is more susceptible to selection bias than a formal systematic review. In addition, case reports were included because of the complexity and relative rarity of several intraoperative scenarios, but these may not be representative of the broader CABG population. Therefore, the findings should be interpreted as a clinically oriented synthesis rather than definitive population-level evidence.

## Figures and Tables

**Table 1:** Summary of included studies and key findings across complex CABG scenarios.

Scenario	Study	Design	Population/Setting	Key Results
Intramural/embedded coronaries	The intramyocardial left anterior descending artery: prevalence and surgical considerations in coronary artery bypass grafting—S Afr J Surg, 2014 [8]	Retrospective cohort	CABG patients (*n* = 1349)	IMLAD present in 21.7% (293/1349); deep septal extension in 3.8% (11/293); emphasizes localization and controlled exposure.
	The Prevalence, Surgical Considerations and Outcomes of Intramyocardial Coronaries in Coronary Artery Bypass Grafting Patients: a 5-year Retrospective Study. European Journal of Cardiovascular Medicine. [9]	Retrospective series	Intramyocardial coronary cases (*n* = 50); prevalence reported as 13%	Describes practical localization signs (vein, tracing, groove, probe) and operative considerations; emphasizes individualized exposure strategy.
	Embedded Left Anterior Descending Artery During Coronary Bypass Operations: A 15-year Experience—Heart Lung Circ, 2015 [10]	Retrospective cohort	CABG patients (*n* = 4102); embedded LAD subgroup (*n* = 92)	Probe-guided localization used in 79% (73/92); ventricular injury in 4 patients (predominantly with direct dissection); no hospital mortality reported in embedded-LAD subgroup.
	Intramyocardial coronary arteries: dissection during coronary artery bypass surgery in 70 patients—Vascular Surgery, 1992 [11]	Cohort-type observational study	Patients with intramyocardial coronaries (*n* = 70)	Deeply embedded vessels increase risk of dissection, ventricular injury, and bleeding during exposure.
	Localizing intramyocardially embedded left anterior descending artery during coronary bypass surgery: literature review—Journal of Cardiothoracic Surgery, 2013 [12]	Literature Review	Review paper of published techniques for localizing intramyocardially embedded LAD during CABG	Summarizes 5 main localization strategies for embedded LAD: retrograde intraluminal probe, retrograde dissection from the apex, Doppler/epicardial ultrasound, intraoperative cineangiography, and tape-elevation techniques. Recommends a stepwise approach starting with surface inspection, then less traumatic imaging methods, then controlled retrograde dissection, with probe-guided exposure as a last resort when depth reaches about 4 mm to reduce RV perforation risk
	Initial Experience with Epicardial Ultrasound Scanning in Coronary Artery Bypass Grafting—Korean Journal of Thoracic and Cardiovascular Surgery, 2020 [13]	Retrospective observational study	CABG patients undergoing routine intraoperative EUS + TTFM (*n* = 53); 141 distal anastomoses, 32 target vessels, and 2 conduit trunks assessed	EUS was used to assess all distal anastomoses and target vessels. Among 18 grafts with abnormal TTFM, only 3 were revised based on EUS, while the other 15 were left unrevised and were all patent at 1 year. EUS also changed the target vessel in 5 patients because of a small-caliber atherosclerotic lumen (<1 mm, n = 2) or an intramyocardial course along the full vessel length (n = 3), supporting its value for target selection and avoidance of unnecessary revision. Early graft patency was 100% and 1-year patency was 96.1%.
	Intramyocardial left anterior descending unroofing using a minimally invasive off-pump approach—Netherlands Heart Journal, 2024 [14]	Case Report	Single 50-year-old male with symptomatic intramyocardial LAD undergoing minimally invasive off-pump unroofing via left mini-thoracotomy	Entire intramyocardial LAD course was successfully unroofed using a minimally invasive off-pump approach; postoperative angiography showed the LAD diameter increased 2–3-fold, recovery was uneventful, discharge occurred after 3 days, and follow-up showed complete remission of chest pain.
Calcified/diffuse disease	Ten-Year Experience of Coronary Endarterectomy for the Diffusely Diseased Left Anterior Descending Artery—Ann Thorac Surg, 2017 [15]	Retrospective cohort	LAD endarterectomy patients (*n* = 188)	30-day mortality 1.1%; periop MI 9.0%; LAD patency 91.6% early and 96.6% at 1 year; 5-year freedom from mortality 89.3%.
	Vein patch angioplasty combined with left internal thoracic artery bypass to left anterior descending artery in patients having diffuse complex atherosclerotic lesions—Medeniyet Medical Journal, 2016 [16]	Retrospective cohort	Patients undergoing SVG patch angioplasty (*n* = 21)	Early mortality 4.8%; survival 90% at 2 years and 81% at 5 years; LITA patency 93.3% among those imaged.
	The Comparison Between Two Surgical Methods for Left Internal Mammary Artery (LIMA) Anastomosis on Left Anterior Descending (LAD) Artery in Patients with Severe Diffuse Lesions: Short to Mid-Term Results—Acta Medica Iranica, 2015 [17]	Randomized trial	Patients with diffuse LAD disease (*n* = 40)	MI occurred only in the long arteriotomy/long anastomosis group; sequential anastomosis associated with greater improvement in LVEF and functional class.
	Vein Patch Angioplasty with Internal Mammary Artery Grafting of the Left Anterior Descending Coronary Artery—Acta Medica Philippina, 2024 [18]	Case series	Patients undergoing SVG patch angioplasty (*n* = 26)	In-hospital mortality 7.7% (2/26); POAF 23.1% (6/26); re-exploration for bleeding 3.8% (1/26).
	Open coronary endarterectomy: a case report and literature review—J Med Life, 2022 [19]	Case report + review	Single patient + literature synthesis	Summarizes operative concepts for open coronary endarterectomy in diffuse/calcified targets.
	Full metal jacket endarterectomy of the LAD: early results and mid-term follow-up—Heart Lung Circ, 2021 [20]	Series/technique report	Patients with full-metal-jacket LAD disease	Frames extensive LAD disease as a reconstruction problem; describes long-segment reconstruction with planned antithrombotic regimen and imaging follow-up.
Small-caliber targets	Safety and efficacy of sequential and composite arterial grafting to >5 branches in off-pump CABG—Eur J Cardiothorac Surg, 2010 [21]	Retrospective cohort	Total-arterial off-pump CABG (*n* = 633; 2617 distal anastomoses)	Patency to targets ≤ 1.25 mm was 97.4% (626/643); bilateral in-situ ITAs associated with higher flow than single ITA configuration.
	1-year outcomes after CABG in patients with target-artery distal calcinosis—Patologiya Krovoobrashcheniya i Kardiokhirurgiya, 2022 [22]	Propensity-matched cohort	CABG patients with distal calcinosis (1-year follow-up)	Small/distally calcified targets required more adjunctive techniques; early ischemic outcomes comparable at 1 year.
	Features and hospital outcomes of coronary artery bypass grafting in patients with calcification of target coronary arteries—Russian Journal of Cardiology, 2020 [23]	Comparative cohort	CABG patients with calcified targets	Calcification and small diameter increased technical complexity and adjunct use without clear early ischemic penalty in matched analyses.
	Patency and adverse outcomes of sequential vs. individual saphenous vein grafts in coronary artery bypass: A meta-analysis—Frontiers in Cardiovascular Medicine, 2022 [24]	Systematic review/meta-analysis	Included comparative SVG studies	Sequential SVGs associated with lower graft failure vs. individual SVGs (RR 0.68).
	Outcomes of single versus sequential vein grafts in isolated coronary artery bypass surgery: Insights from a large tertiary care center—Journal of Thoracic and Cardiovascular Surgery, 2025 [25]	Retrospective cohort	Isolated CABG patients	Sequential vs. single vein grafting showed no significant differences in early/late patency or short/long-term mortality.
	Transit-Time Flow Measurement and outcomes in CABG patients—Semin Thorac Cardiovasc Surg, 2023 [26]	Observational study	CABG patients undergoing TTFM	Lower mean graft flow and higher pulsatility index associated with inferior patency; PI > 5 independently predicted MACCE.
	Impact of intraoperative TTFM on off-pump and on-pump CABG—Heart Surg Forum, 2024 [27]	Propensity score–matched study	CABG patients (3-month assessment)	TTFM identified grafts with suboptimal flow characteristics and prompted revision in a subset; supports intraoperative quality control.
Iatrogenic RV injury	Sandwich technique for iatrogenic RV injury during beating-heart CABG—Heart Lung Circ, 2020 [28]	Two-case report	Beating-heart CABG cases (*n* = 2)	Sandwich technique enabled hemostasis without compromising LAD/graft flow; both discharged around POD 5.
	Management of ventricular perforation during revascularization of an intramyocardial left anterior descending artery—Journal of Cardiac Surgery, 2017 [29]	Case report	Single patient	4 mm RV defect repaired; preserved graft flow (22 mL/min; PI 2.2); discharged POD 7; CCS class I at 18 months.
	Secure Closure of Right Ventricular Perforation Reinforced With Coronary Artery Wall—Annals of Thoracic Surgery, 2020 [30]	Technical note/case report	Single patient	Adds coronary wall reinforcement to distribute suture tension and reduce rebleeding while preserving luminal/graft integrity.
	Management of right ventricular injury after localization of the left anterior descending coronary artery—Annals of Thoracic Surgery, 2009 [31]	Technical Note	Operative technique description/illustrative cases	Highlights mechanisms of injury and practical closure aids designed to protect the LAD during repair.
	Clipless internal mammary artery harvesting for minimally invasive coronary artery bypass grafting using the shear-tip harmonic scalpel—Journal of Thoracic Disease, 2024 [32]	Retrospective cohort	Patients undergoing minimally invasive CABG (*n* = 40)	Clipless skeletonized IMA harvesting with a shear-tip Harmonic scalpel was feasible and yielded stable early results: graft patency was achieved in all patients, 38/40 cases were completed without cardiopulmonary bypass, no graft-related complications were reported, all patients were discharged uneventfully, and only 1 late graft occlusion required intervention during median 15.2-month follow-up.
Failed PCI/stent-related	Surgical approaches in left anterior descending artery in-stent stenosis—Annals of Thoracic Surgery, 2008 [33]	Case Series	Patients with long-segment LAD in-stent stenosis undergoing surgical treatment (*n* = 7)	Included 7 patients with prior LAD stenting treated using stent removal and coronary endarterectomy; 2 cases were minimally invasive off-pump and 5 were performed with cardiopulmonary bypass. Postoperative coronary angiography was performed in all patients. Authors concluded that stent removal with coronary endarterectomy was a safe and effective strategy for long-segment LAD in-stent stenosis.
	Off-pump coronary endarterectomy with stent removal for in-stent restenosis in the left anterior descending artery—Interactive Cardiovascular and Thoracic Surgery, 2015 [34]	Technical report/small series	Patients with long-segment LAD in-stent restenosis undergoing OPCAB stent removal + endarterectomy (*n* = 12)	Demonstrates feasibility of off-pump stent removal + endarterectomy with angiographic follow-up supporting reconstructed target suitability.
	Early and Midterm Results of Stent Endarterectomy for Left Anterior Descending Coronary Artery “Full Metal Jacket”—Heart Surgery Forum, 2021 [35]	Clinical series	Patients with full-metal-jacket LAD disease	Controlled reconstruction (stentectomy + endarterectomy + long reconstruction) with planned antithrombotic regimen and follow-up.
	Coronary endarterectomy combined with coronary artery bypass grafting might decrease graft patency: A cohort study—Hellenic Journal of Cardiology, 2024 [36]	Cohort study	CABG with angiographic follow-up (*n* = 160)	Overall patency to endarterectomized targets 69.9%; LAD-CE 80.0% vs. non-LAD 64.2%; postop MI 4.4%; RCA-CE predicted graft failure (OR 2.35).
	Coronary Stent Infection: A Systematic Review of Literature—Cardiology in Review, 2025 [37]	Systematic Review	1 case series and 41 case reports; cumulative sample size of 44 patients with coronary stent infection	Coronary stent infection was identified as a rare but severe complication with an aggregate mortality of 18%. Morbidity ranged from 3% to 60%, with complications including sepsis, heart failure, and embolic events; recurrence ranged from 3% to 33%. Management strategies varied widely, including antibiotics alone, antibiotics with stent removal, or antibiotics with stent retention, underscoring the lack of standardized treatment and the importance of early diagnosis and operative contingency planning.
	Drug-coated balloon angioplasty, intraoperatively through left anterior descending arteriotomy access, a novel hybrid revascularization strategy: a case report—European Heart Journal Case Reports, 2023 [38]	Case report	Single patient	Describes hybrid bailout revascularization using intraoperative DCB angioplasty through LAD arteriotomy access.

**Table 2:** Early angiographic patency by target-vessel diameter in bilateral in situ internal thoracic artery off-pump CABG: Subgroup II-A (3–4 distal anastomoses) vs. Subgroup II-B (≥5 distal anastomoses) [21].

Target-Vessel Diameter	Subgroup II-A Patency	Subgroup II-B Patency
1.0 mm	14/15 (93.3%)	49/52 (94.2%)
1.25 mm	151/156 (96.8%)	222/224 (99.1%)
≥1.5 mm	428/436 (98.2%)	498/506 (98.4%)

## Data Availability

No new data were created or analyzed in this study. Data sharing is not applicable to this article.
